# Bioavailability of inhaled or ingested PFOA adsorbed to house dust

**DOI:** 10.1007/s11356-022-20829-3

**Published:** 2022-06-14

**Authors:** Åsa Gustafsson, Bei Wang, Per Gerde, Åke Bergman, Leo W. Y. Yeung

**Affiliations:** 1 MTM Research Center, School of Science and Technology, Örebro University, SE-701 82 Örebro, Sweden; 2 Institute of Environmental Medicine, Karolinska Institutet, SE-171 77 Stockholm, Sweden; 3 Inhalation Sciences AB, Hälsovägen 7-9, SE-141 57 Huddinge, Sweden; 4 Department of Environmental Science, Stockholm University, SE-106 91 Stockholm, Sweden

**Keywords:** Perfluorooctanoic acid, Household dust, Ingestion, Gastro intestinal (GI), Airways, Adsorption, PFAS

## Abstract

Indoor environments may impact human health due to chemical pollutants in the indoor air and house dust. This study aimed at comparing the bioavailability and distribution of PFOA following both an inhalation and an oral exposure to PFOA coated house dust in rats. In addition, extractable organofluorine (EOF) was measured in different tissue samples to assess any potential influence of other organofluorine compounds in the experimental house dust. Blood samples were collected at sequential time points after exposure and at the time of termination; the lungs, liver, and kidney were collected for quantification of PFOA and EOF. The concentration of PFOA in plasma increased rapidly in both exposure groups attaining a C_max_ at 3 h post exposure. The C_max_ following inhalation was four times higher compared to oral exposures. At 48 h post exposure, the levels of PFOA in the plasma, liver, and kidney were twice as high from inhalation exposures. This shows that PFOA is readily bioavailable and has a rapid systemic distribution following an inhalation or oral exposure to house dust coated with PFOA. The proportion of PFOA to EOF corresponded to 65–71% and 74–87% in plasma and tissues, respectively. The mass balance between EOF and target PFOA indicates that there might be other unknown PFAS precursor and/or fluorinated compounds that co-existed in the house dust sample that can have accumulated in rats.

## Introduction

Perfluorooctanoic acid (PFOA) belongs to the large group of per and polyfluoroalkyl substances (PFASs) which include more than 4700 substances (OECD 2018; Wang et al. 2017). Due to the unique properties of PFAS being both water and grease repellent, they have been used in numerous industrial and commercial products (DeWitt et al. 2019). Most PFASs, including PFOA, are extremely resistant to any chemical, physical, or biological transformations; they are highly persistent and ubiquitously found in different environmental compartments (Buck et al. 2011; De Silva et al. 2021). Higher bio-accumulative potentials were observed for those PFAS having more than five fluorinated carbons for sulfonates and more than seven fluorinated carbons for carboxylates (DeWitt et al. 2019; Stahl et al. 2011).

The bioavailability of PFOA, crossing biological barriers, is indicated by the detection of PFOA in blood and mothers’ milk samples (Awad et al. 2020; Calafat et al. 2007; Tao et al. 2008; Völkel et al. 2008; Ye et al. 2018). The major route of exposure to PFOA in humans comes from ingestion of contaminated food and drinking water (Hu et al. 2019; Sadia et al. 2020; Stahl et al. 2011; Trudel et al. 2008). In addition, other exposure pathways to PFOA have been identified via indoor air and dust through inhalation, ingestion, and dermal uptake (Salthammer et al. 2018). Haug and colleagues demonstrated that the indoor environment is an important factor of human exposure to PFAS like PFOA (Haug et al. 2011a). PFOA and its derivatives are found in consumer products like, e.g., carpets and furnishing, cookware, food papers, and clothing (Egeghy and Lorber 2011; Goosey and Harrad 2011; Verner et al. 2015). The presence of PFOA on house dust is ubiquitous from all over the world (Björklund et al. 2009; Byrne et al. 2017; D'Hollander et al. 2010; Eriksson and Kärrman 2015; Goosey and Harrad 2011; Haug et al. 2011b; Huber et al. 2011; Kato et al. 2009; Knobeloch et al. 2012; Kubwabo et al. 2005; Moriwaki et al. 2003; Schoeib et al. 2016; Xu et al. 2013; Tian et al. 2016; Weiss et al. 2021; and Winkens et al. 2018). Considering that PFAS are extensively used in indoor products and owing to the long time that we spend indoors daily (approximately 90%), it is of great importance to determine the bioavailability of PFOA from house dust via relevant exposure pathways. The bioavailability over the air-blood-barrier following inhalation of aerosolized PFOA to rats has indicated on a high uptake over the airways (Hinderliter et al. 2006). Also, the absorption of PFOA (ammonium salt: APFO) following an oral exposure in animal studies showed that virtually all of it was absorbed over the gastrointestinal tract (GI) (DeWitt. 2015). The investigation of the bioavailability of PFOA from inhaled house dust has to our knowledge never been reported before.

Fluorine mass balance analysis between extractable organofluorine (EOF) and quantified PFAS has been used to indicate the presence of unidentified organofluorine compounds (Koch et al. 2019). In the current study, we chose PFOA for investigation of bioavailability following exposure to house dust; previous studies showed the presence of different classes of PFAS in indoor house dust (Eriksson and Kärrman 2015; Winkens et al. 2018; Weiss et al. 2021; Poothong et al. 2019). Fluorine mass balance analysis in current study may demonstrate the presence of other unidentified PFAS and metabolites as well as conjugated products formed upon exposure.

The objectives of this study were to investigate the bioavailability of PFOA in rats following inhalation or ingestion of house dust. High amounts of PFOA were adsorbed to the surface of a respirable fraction of house dust. A relevant inhalation exposure condition was established by using the PreciseInhale system, where intubated rats inhaled house dust spontaneously. The levels of PFOA were then analyzed in plasma at several subsequent time points. The time kinetic of PFOA levels in the plasma was compared between the inhalation and oral exposure to house dust. Also, the distribution of PFOA to different tissues was compared between the two exposure pathways. In addition, this study investigated the amounts of unidentified organofluorine compounds in plasma and different tissue compartments.

## Materials and methods

### Chemicals

The PFOA (≥ 98%) for dust coating experiments was purchased as solid standard from Sigma-Aldrich (St. Louis, MO, USA). Analytical native standard of PFOA (perfluoro-n-octanoic acid), mass-labelled extraction standard served as internal standard (IS), perfluoro-n-[1.2.3.4- 13C4]-octanoic acid), and mass-labelled injection standard served as recovery standard (RS), perfluoro-n-[ ^13^C8]-octanoic acid), were purchased from Wellington Laboratories (Guelph, Ontario. Canada). Ammonium acetate, methyl-*tert*-butyl ether (MTBE) (≥ 99.8%), tetrabutylammonium bisulfate (TBA) with a purity of ≥ 99%, and methylpiperidine (1-MP, 99%) were purchased from Sigma-Aldrich® (St. Louis, MO, USA). OASIS® WAX (6 mL, 150 mg) was from the Waters Corporation (Milford, MA, USA). Formic acid (> 95%), methanol HPLC-grade (≥ 99.8%), methanol LC-MS-grade (≥ 99.9%), acetonitrile HPLC-grade (≥ 84 99.9%), and ammonium solution (25%) were purchased from Fisher Scientific (Pittsburgh, PA. USA).

### Respirable fraction of house dust

The house dust was obtained from vacuum cleaner bags collected from 32 residential homes in the region of Stockholm, Sweden. The respirable fraction of house dust was retrieved by a sieving process of which all 32 vacuum cleaner bags were pooled. The processing of dust, to a respirable particulate size fraction, has been described in detail by Gustafsson et al. (2018). Briefly, the dust was sieved through six stages of plane woven steel meshes, followed by passage of a cyclone, after which the dust was collected in a filter bag. The dust was once again sieved through a twilled woven steel, yielding the respirable fraction, here defined as a size fraction below < 5 μm. The sieving process was performed under constant airflow and mechanical deagglomeration.

### Sorption of PFOA on house dust in the gas phase

To achieve a sufficient concentration of PFOA on the house dust, a saturated vapor concentration procedure was created to adsorb/absorb PFOA on the house dust. This was done to ensure detectability in the tissue samples. Ten grams of solid pure perfluorooctanoic acid was placed in a glass jar bottle, capped with a lid, shaken and stored at room temperature (25 ± 0.1 °C) to allow vaporization and pre-equilibration of PFOA on the walls of the glass jars. Following equilibration of PFOA in the air and on walls of jars, a portion of 5 ± 0.03 g dust was placed in a polypropylene (PP) round plate inside each of four jars. These were capped and kept under the controlled room temperature and atmospheric pressure over time, mimicking a residential indoor environment, but with a saturated vapor of PFOA. Repeated samples of dust were withdrawn from the jars over time and analyzed for the PFOA content. This procedure was repeated over time for up to 6 weeks until a stable equilibrium concentration of PFOA was attained adsorbed to the dust. The long equilibration time in the near saturated gas-phase of PFOA allowed for an even adsorption/absorption on the dust particles in the testbed.

During the adsorption process, the concentration of PFOA adsorbed to the dust was periodically measured after a rotatory mixing of dust samples with a tube rotator or 24 h prior to the extraction procedures and instrumental analysis. Preliminary trials showed that this rotatory mixing prevented the formation of pockets of condensed PFOA at the outer edges of the sieved dust facing higher air concentrations of PFOA. The procedure resulted in a uniform adsorption over the entire dust sample on the PP plate. Measurements were repeatedly performed until a saturation of PFOA was reached on the dust. At the time when the concentration was within 10% of the previous measurement, the dust was considered saturated. The dust was stored in a −20 °C freezer until the animal exposure experiment. Four replicated dust adsorption tests were prepared identically in this manner to verify the reproducibility of the dust adsorption experiment and homogeneity of the PFOA adsorbed dust samples.

### Animals

This study was conducted in accordance with a protocol approved by the Animal Committee of ethics in Linköping, Sweden according to Directive 2010/64/EU.

Male rat RccHan:WIST, approximately 22 weeks of age (450–575 g), were purchased from Envigo, Venray, Netherlands. The rats were housed five each in clear polycarbonate cages (20 × 25 × 47 cm) containing enrichment such as nesting material, chew sticks, and tunnels. Water from the municipal tap and extruded rodent diet 2016 Teklad global, 16% protein (Envigo. Venray. Netherlands) were provided ad libitum. Animals were housed in a facility that maintained an average temperature of 23.2 °C (Min: 21.6–Max: 23.9), average humidity of 51.1% (Min: 25.4–Max: 60), and a 12 h light:12 h dark cycle.

### Experimental design

A total of 10 animals were used in the study. Four rats in each group were exposed by either inhalation or gavage, respectively. Two rats were unexposed and used as experimental control animals. For both exposed groups, the blood samples were collected prior to exposure (0 h) and then at 3, 6, 24, and 48 h post exposure. Blood samples were collected (a volume of ~200 μl) from the tail vein on wake rats. The rats had been habituated to the sampling method before the study in order to minimize stress (refine). At the time of termination, 48 h after exposure, the rats were exsanguinated under isoflurane and oxygen anesthesia. At the time of termination, the blood was collected during the exsanguination. The blood samples were collected in microvette 200z (Sarstedt). The blood was kept on ice after collection. The blood samples were centrifuged at 2000 *g* for 5 min and the plasma samples were stored at −20 °C until the analyses. Bronchoalveolar lavage fluid (BALF) was collected with 5 mL of sodium chloride by cannulating the trachea and flushing gently back and forward twice before sample collection. The sample was stored in a freezer at −20 °C until the analysis. The lungs, liver, and kidney were removed and frozen in liquid nitrogen followed by freezer storage (−20 °C) until analyses. All plastics that were used were made of PP or high-density polyethylene to avoid loss of PFOA due to adsorption to the plastics.

### Aerosol generation and aerodynamic particle size distribution

An aerosol was generated with the PreciseInhale® platform (Inhalation Sciences Sweden AB, Stockholm, Sweden) and particle size distribution of the house dust was measured with a nine-stage Marple cascade impactor (Marple and McCormack 1983). The dust was aerosolized batch wise into a 300 mL holding chamber of the PresiceInhale® and then pushed out at a flow rate of 330 mL/min. Prior to aspiration into the impactor, the aerosol was diluted into a continuous airflow of 2 L/min. The mass of dust deposited on the nine stages in the impactor was used to calculate the mass median aerodynamic diameter (MMAD) and the geometric standard deviation (GSD). The method was adapted from Selg and co-workers (Selg et al. 2010; 2013) and the aerodynamic characterization of the respirable fraction of the pooled house dust used in this study has previously been described by Gustafsson et al. (2018).

### PFOA dust inhalation exposure

The house dust was aerosolized with the DustGun powder generator and then delivered to the rats with the PreciseInhale dispensing system. In order to determine the system settings for reaching the target dose of dust, pre-exposure filter experiments were performed with the PreciseInhale system. The inhaled mass of the dust collected using an in vitro filter test system for rats exposed in vivo by intratracheal intubation was calibrated against the optical signal from a Casella Microdust Pro light dispersion instrument (Casella CEL, Inc., Buffalo, NY). An inhaled dose of approximately 0.5 mg of dust per animal was decided, to compare to 0.5 mg for the gavage. The achievable dose of 0.5 mg house dust inhaled resulted in a deposited dose of 0.26 mg per rat, based on a calculated deposition fraction of 0.51 as determined with the MPPD model for the particle size distribution of the house dust (Price et al. 2002). Because of a time limitation on how long the animals could be kept intubated, the previously achieved deposited dose by gavage could not be fully reached via inhalation. However, for organic inhalants such as PFOA, dose normalization between adjacent exposure levels can be accurately achieved with retained accuracy of the pharmacokinetics (Malmlöf et al. 2019).

The rats were anesthetized by administrating a premedication of 0.05 mg/mL atropine (50 μg/kg bw) subcutaneous followed by a cocktail of fentanyl (10–15 μg/kg bw) and Dormitor Vet. (0.21–0.30 mg/kg bw) (50:50) that was administrated gradually in the tail vein intravenous until anesthesia was achieved. The rats were intubated with a stainless-steel catheter (outside diameter 2.02 mm, inside diameter 1.67 mm, length 6 cm) using a laryngoscope. The intubated rat was placed on heating pad in supine position on an adjustable table and connected to the PreciseInhale system. Each animal was monitored for 5 min before aerosol exposure to ensure stable spontaneous breathing. Four animals were exposed to 9–10 exposure shots of dust during an exposure time of approximately 10 min. The generated aerosol was drawn from the aerosol holding chamber past the breathing animal at a superimposed flow rate of 340 mL/min, which was close to the optimal relation between ventilation rate and the superimposed flow rate for wasting a minimum of test substance, yet preventing rebreathing of exhausted aerosol (Moss et al. 2006). After the inhalation exposure, the rats were injected intramuscularly with Naloxon (0.08–0.1 mg/kg bw) for a fast recovery from anesthesia.

### PFOA dust oral gavage exposure

A suspension of 0.5 mg of dust was prepared in 2 mL tap water. The rats were gavaged by a single dose of dust with PFOA. After the delivery of dust, the tube was rinsed with an additional volume of 1.5 mL of tap water which was also delivered to the rats.

### Chemical analysis

#### Gas phase PFOA sampling and analysis

The gas phase of PFOA was collected with OSHA Versatile Sampling (OVS) tubes with XAD-2 resin (SKC). Samples were collected at a flowrate of 0.04 L/min using vacuum sampling pumps. Collection times were set for 5 min typically for a 200 mL air sample. PFOA from the OVS sampler was extracted prior to analysis into two fractions with each fraction placed into individual PP tubes. Fraction A consisted of the glass or quartz fiber filter and first section of XAD resin beads and the first polyurethane foam (PUF) filter. Fraction B consisted of the second section of XAD resin beads and the back PUF filter. All samples were spiked with 50 μL of 1 μg/mL C13 solution, the surrogate standard. Each section was placed in PP tube to which 5 mL of HPLC-grade methanol was added. The tube was shaken for 60 min on a mechanical flatbed shaker set at ∼2 cycles per second. Approximately 4.5 mL of each extract was transferred to a pre-baked amber glass vial by filtering through a Gelman GHP Acrodisc (Pall Gelman Laboratory, Ann Arbor, MI, USA.). Each extract of consisting of 200 μL was transferred to an autosampler vial with PP insert. After that, 10 μL of recovery standard was added to the autosampler vial for PFAS analysis using LC-MS/MS.

#### Respirable fraction of dust

Internal standard (50 μL) was added to the sieved dust (50 mg) and was left to equilibrate overnight. The sample was extracted with methanol (2 mL) with 30 s vortex and 15 min sonication in-between. The extract was centrifuged (3000 rpm) for 10 min and the supernatant transferred into a new tube. The procedure was repeated once with methanol (1 mL). The combined extract for PFOA analysis was cleaned up by diluting it in 25 mL MilliQ water and the sample was adjusted to pH 10 with ammonium hydroxide. Hexane (4 mL) was added to the water, separated by centrifugation (3000 rpm) and the hexane phase was discarded. The pH of the water was adjusted to pH 4 by adding formic acid. The extract was added to a WAX cartridge (Oasis WAX 150 mg 6cc. Waters), washed with ammonium acetate (pH 4) and tetrahydrofuran (Biosolve)/methanol (3/1), and the analyte eluted with methanol 0.1% ammonium hydroxide. The eluate was evaporated to dryness and reconstituted in 40% methanol in ammonium acetate (2 mM).

#### Plasma and lung lavage

The procedure for extraction of plasma samples followed published method (Yeung et al. 2009) with some modifications. Briefly, 10 μL of mass-labelled internal standard was spiked in to the 50 μL diluted plasma samples (in MilliQ water); they were mixed with 1 mL of 0.5M TBA solution in a 15 mL PP tube. After mixing, 5 mL of MTBE was added, and the mixture was shaken for 20 min at 250 rpm. The organic and aqueous layers were separated by centrifugation at 3000 rpm for 15 min. The organic layer MTBE (4 mL) was transferred to a new PP 15 mL tube. The extraction was repeated twice with 5 mL of MTBE was removed each time. The three extracts were combined in the second PP tube. A 1 mL of methanol was added to the final extract before it was concentrated to 1 mL under nitrogen. The extract was further concentrated to 200 μL in a LC vial, and mass-labelled recovery standards were spiked into the vial. Aqueous ammonium acetate (2 mM, 300 μL) was added to the vial for PFAS analysis using LC-MS/MS.

#### Tissues

All tissue samples were homogenized using a Tissue Tearor (BiospecProducts) with tissue (1 g wet weight) and 1% potassium chloride solution (0.2 mL); the homogenate was then spiked with 10 μL of mass-labelled internal standard before the ion-pairing extraction procedure as described above for the plasma samples.

#### Extractable organofluorine (EOF) analysis

Plasma and tissue samples taken at 48 h after exposure were subjected to EOF analysis. Extraction procedure followed the procedure described above with the exception that no mass labelled internal standards were spiked to the samples before extraction. The EOF content of the 1 mL extract was analyzed by combustion ion chromatography (CIC). Part of the extract (200 μL) was transferred to a LC vial with the addition of 10 μL of mass-labelled standards; after that, aqueous ammonium acetate (2 mM, 300 μL) was added to the vial for PFAS analysis using LC-MS/MS. The reported EOF concentrations as well as the concentration of PFOA for mass balance analysis were not recovery-corrected.

#### Instrumental analysis of PFOA

PFOA was analyzed using a Waters Acquity UPLC coupled to a Waters Xevo TQ-S triple quadrupole mass spectrometer operating in negative ion mode for electrospray ionization. A Waters Acquity UPLC BEH C18 column (1.7 μm, 50 × 2.1 mm, Waters) was heated to 50 °C with a flow rate of 0.5 mL/min. A 10 μL extract aliquot was injected onto the column with mobile phases consisting of 2 mmol/L ammonium acetate in a mixture of MeOH and water MeOH 30/70 (v/v) (A) and 2 mmol/L ammonium acetate in MeOH (B). Details of the LC-MS method are presented elsewhere (Aro et al. 2021).

#### Instrumental analysis of extractable organofluorine (EOF)

Levels of EOF in the sample extracts were determined with a combustion ion chromatography (CIC) system consisting of a combustion module (Analytik Jena, Germany), a 920 Absorber Module and a 930 Compact IC Flex ion chromatograph module (both from Metrohm, Switzerland). An ion exchange column (Metrosep A Supp 5–150/4.0), with carbonate buffer (64 mmol/L sodium carbonate and 20 mmol/L sodium bicarbonate) as the mobile phase, was used for the separation of anions; the absorber solution was water. The sample extract (100 μL) was set on a silica boat via an autosampler and placed into a furnace at 900–1000 °C. The combustion of the sample in the furnace converted organic fluorine and inorganic fluorine into hydrogen fluoride (HF), which was then trapped by MilliQ water. The fluoride concentration in the solution was analyzed using ion chromatography. A five-point calibration curve at 50, 100, 200, 500, and 1000 ng/mL PFOS standards was constructed using the combustion method as samples and exhibited good linearity with *R*^2^ > 0.9999. Quantification was based on external calibration. The analytical conditions for ion chromatography have been reported elsewhere (Kärrman et al. 2021).

#### Mass balance analysis approach

The measured PFOA concentration (ng/mL) in the sample extract of the EOF analysis was converted to the corresponding fluoride-equivalent concentration (ng/mL F) using the following formula:

1 $${\mathrm{CF}}_{\mathrm PFAS}=\mathrm{CPFAS}\ast\mathrm{NF}\ast\mathrm{AF}/\mathrm{MWPFAS}$$

where CPFAS is the concentration of the target compound (i.e., PFOA), NF is the number of fluorine atoms in the target compound (i.e., 15), AF is the atomic weight of fluorine (g/mol, i.e., 19), and MWPFAS is the molecular weight of the target compound (i.e., 413). The sum of known extractable fluorine concentration (ΣCF_PFAS) was calculated by summing the fluorine concentrations from all individual PFASs. Values below limits of quantification (LOQ) were set for calculating ΣCF_PFAS. Levels of unidentified organofluorine were calculated by subtracting EOF from all quantifiable PFAS, which in this present study only PFOA.

#### Quality assurance and quality control (QA/QC)

In PFOA analysis, the method detection limits (MDLs) were determined as three times the signal in the negative control; and in absence of the analyte in the blank the lowest point in the calibration curve, which ranged 0.02–0.04 ng/mL for most of the PFOA. To ensure stable sensitivity over the entire instrumental analysis, a quality assurance (QA) sample made of PFOA standards (2 ng/mL) was injected between each eight samples; the relative standard deviation of the intensity of QA samples was found to be below 10%. Before real sample analysis, matrix spike recoveries were conducted by spiking 1 ng of PFOA into different tissues (e.g., plasma, liver, lungs, lung lavage, and kidney) of the control subjects, and the accuracy of the method was evaluated by subtracting the spiked level from the non-spiked sample and then divided by the spiked level times 100%; results were found to be between 92 and 111% (Table S1, Supplementary Information).

For the analysis of EOF, multiple measurements of combustion blanks were effectuated and repeated until the combustion blanks showed low variability (below 5% relative standard deviation) over the last three combustion blanks, so as to reduce the CIC system contained background fluoride contamination. All measurements of samples were first subtracted from the combustion blanks between samples before quantification, using an external calibration curve. An instrumental standard (PFOS 480 ng F/mL) was analyzed to evaluate the whole performance of the CIC. Signal fluctuation (RSD: 15%) was observed in the instrument standard in every five samples. A spiked plasma sample containing 200 ng F/mL was extracted in triplicate and the recovery was found to be 80 ± 9% and the relative standard deviation was found to be 8%.

## Results

### Vapor concentration of PFOA

The vapor concentration of PFOA (Cpfoa) measured at the outlet of the vapor pressure generation chamber is described in detail in Supplementary Information and shown in Fig. S1. These measurements were made after 1, 3, 12, 24, and 48 h at room temperature. Prior calculations showed that a complete evaporation of the coating (a few grams) at the designated emission rates would suffice for the duration of the test (48 h) based on the vapor pressure of PFOA. A stable vapor concentration indicating saturation was established already after 24 h at a concentration of 13.7 × 10^−6^ (μg/L) (Fig. S1), and this is the point we used as reference time point in the adsorption of PFOA on the dust (see next section).

### Sorption of PFOA on house dust

**Fig. 1:**
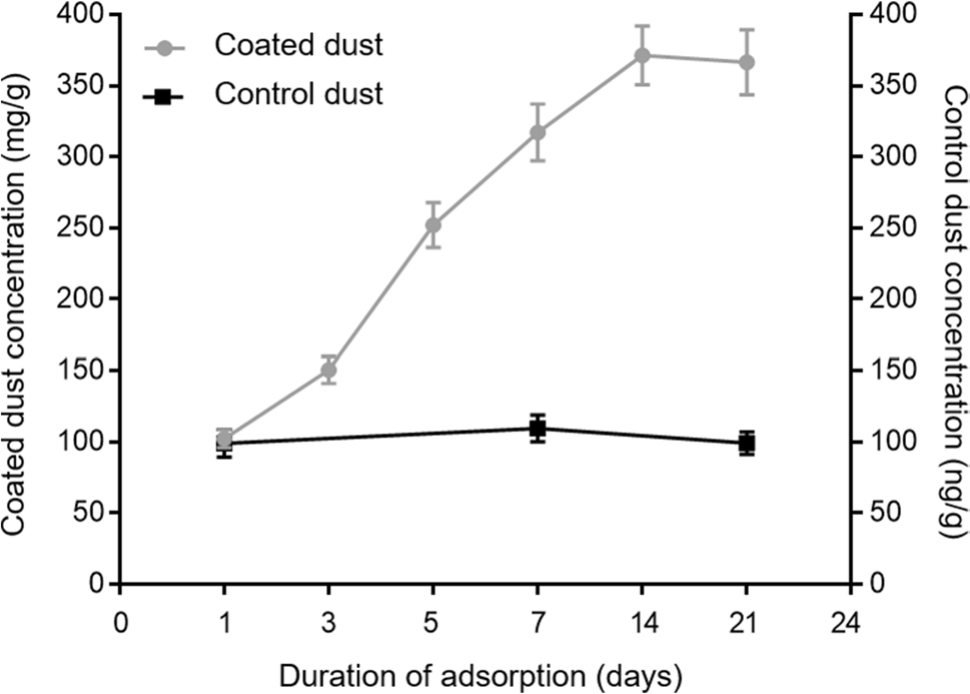
The concentrations of reference-coated dust throughout the whole dust-coated experiment and homogeneity sample analyses between replicated adsorption experiments (*n* = 3)

After equilibrating in a near saturated vapor of PFOA for more than 3 weeks, the house dust reached a steady state concentration of PFOA of 0.335 g/g coated dust (Fig. 1). This likely represents both a smaller fraction of true surface adsorbate and a dominating fraction of absorbed PFOA, dissolved in the more or less liquid constituents of the dust. In addition, some PFOA may have formed condensate in the porous structures of the dust. If the surface adsorbate of monomolecular PFOA has about the same surface density as for aromatic hydrocarbons, or 0.0004 g/m^2^ (Gerde et al. 2001), the fully coated BET surface area of the dust of 2.5 m^2^/g would correspond to a dust concentration of only 0.001 g/g. Therefore, the dust content of PFOA is likely dominated by absorbed fractions and capillary condensates.

### Aerosol generation and inhalation exposures

**Fig. 2:**
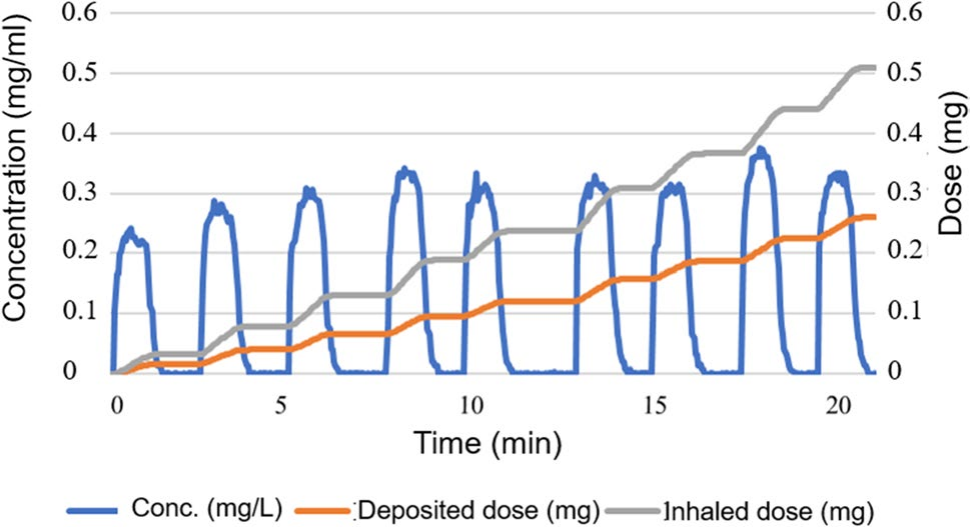
The aerosol concentration and accumulated doses of house dust in one of the intratracheally intubated rats during one complete exposure session. The exposure required 9 consecutive aerosol generation cycles of the PreciseInhale system. The blue line shows the aerosol concentration during the 9 consecutive aerosol generation cycles. The grey line shows the cumulative inhaled mass of aerosolized house dust as calculated by the dose counter of the PreciseInhale system based on measured ventilation rate and aerosol concentration during the exposure. The orange line displays the deposited dose when the inhaled dose has been adjusted for a 50% deposition fraction of the exposure aerosol, as calculated from the theoretical MPPD model

For the inhalation exposures of the rat, an aerosol of house dust was generated, with a MMAD and GSD of 3.7 μm and 2.3, respectively, as published in our previous study (Gustafsson et al. 2018). The intratracheally intubated lungs of the four rats were exposed to reach an inhaled mass of 0.51 ± 0.03 mg of aerosolized house dust according to the real time dose counter of the PreciseInhale system. The aerosol concentration during the exposure cycles ranged between 0.25 and 0.35 mg/L (Fig. 2). The aerosol was delivered to each exposed rat during 20–25 min, requiring 9–10 reloading’s of house dust in order to reach the desired deposited mass of 0.26 ± 0.01 mg (Fig. 2).

### Delivered dose of PFOA

The dose of PFOA was calculated based on the mass of PFOA coated on the house dust (335 mg PFOA/g dust) and the deposited dose administrated to each group. The deposited dose following inhalation or oral gavage of dust resulted in a PFOA dose of 164 μg PFOA/kg BW and 364 μg PFOA/kg BW, respectively.

### PFOA in plasma samples

**Fig. 3:**
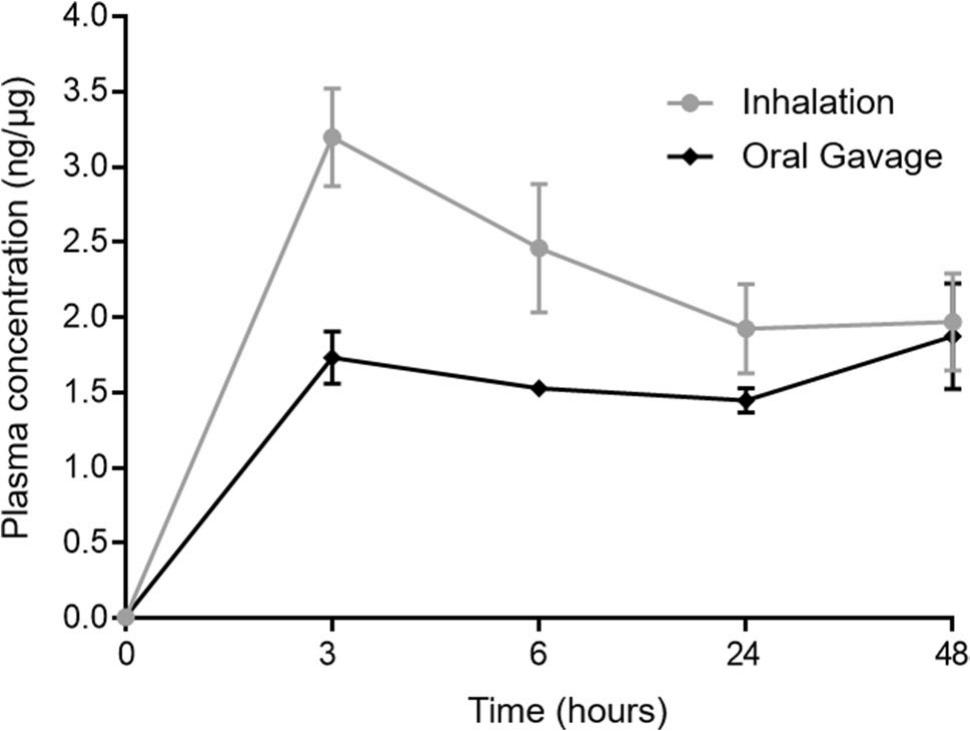
Concentration (ng/μl) of PFOA over time in plasma from rats following inhalation and gavage administration of house dust coated with PFOA (*n* = 4). At time zero, the level of PFOA was 0.01 ± 0.002 ng/μl (*n* = 10)

Both the inhalation and gavage groups showed a rapid increase of PFOA concentrations in plasma following exposure (Fig. 3). The C_max_ occurred at 3 h post exposure with a value of 3.10 ± 0.32 ng/μL in the inhalation group (*n* = 4) and 1.73 ± 0.17 ng/μL in the oral gavage group (*n* = 4). In the group that received dust by inhalation, the concentration of PFOA decreased at 6 and 24 h post exposure, followed by a steady state at 48 h post exposure (Fig. 3). For the group that received dust by gavage, there was an increase of PFOA in plasma until 3 h post exposure followed by a steady state of the concentration of PFOA in plasma for the remaining sampling times at 6, 24, and 48 h post exposure (Fig. 3).

### PFOA in lung lavage, terminal plasma and tissues

The concentrations of PFOA were measured at 48 h after dust exposure in the lung lavage, lung tissue, liver, and kidney (Table 1). For the group receiving PFOA by inhalation, the largest proportion of PFOA was measured in the lung tissue (31.3 ± 4.00 μg/g) followed by the liver (10.1 ± 0.88 μg/g), lung lavage (9.98 ± 0.63 ng/μl), and kidney (3.84 ± 0.76 μg/g). For the group receiving PFOA by gavage, the largest proportion was measured in the liver (8.94 ± 0.50 μg/g), followed by the kidney (3.15 ± 0.19 μg/g), lung (1.68 ± 0.31 μg/g), and lung lavage (0.31 ± 0.02 ng/μl). Similar concentrations of PFOA were measured in plasma for inhalation and gavage exposures with concentrations of 2.03 ± 0.32 ng/μL and 1.87 ± 0.35 ng/μL, respectively (Table 1). The ratios of PFOA concentration between liver and plasma were 5.28 ± 1.3 and 4.89 ± 1.0 following inhalation and oral exposure, respectively. Similarly, the ratio of PFOA concentration between kidney and plasma was determined to be 2.02 ± 0.7 and 1.72 ± 0.3 for inhalation and oral exposure, respectively (Table 1).

**Table 1:** Mean PFOA and PFOA-fluorine equivalent concentrations (ng/μl F or μg/g F) of measured PFOA and extractable organofluorine (EOF) in terminal plasma, lung lavage, and organs sample

Matrix	PFOA^1^		PFOA-fluorine equivalent^2^		EOF^2^		Contribution to EOF (%)	
	Inhalation	Oral	Inhalation	Oral	Inhalation	Oral	Inhalation	Oral
Plasma	1.97 ± 0.32	1.87 ± 0.35	1.53 ±0.17	1.42 ±0.24	2.15 ±0.03	2.18 ±0.13	71	65
Lung lavage	9.98 ± 0.63	0.31 ± 0.02	6.87 ± 0.43	0.21 ± 0.02	9.01 ± 0.67	0.25 ± 0.02	76	84
Liver	10.1 ± 0.9	8.94 ± 0.5	6.95 ± 0.65	6.14 ± 0.34	7.97 ± 0.69	7.36 ± 0.36	87	83
Lung	31.3 ± 4.0	1.68 ± 0.3	21.5 ± 2.75	1.16 ± 0.21	24.9 ± 2.76	1.55 ± 0.28	86	74
Kidney	3.84 ± 0.8	3.15 ± 0.2	2.64 ± 0.53	2.16 ± 0.13	3.26 ± 0.65	2.63 ± 0.18	81	83
Liver:plasma^a^	5.28 ± 1.3	4.89 ± 1.0						
Kidney:plasma^a^	2.02 ± 0.7	1.72 ± 0.3						

^1^Concentration is present in ng/μl for plasma and lung lavage and μg/g for organ samples. ^2^Concentration is present in ng/μl F for plasma and lung lavage and μg/g F for organ sample. ^a^Ratio

### Extractable organofluorine (EOF)

Plasma and tissue samples taken at 48 h after exposure showed detectable EOF concentrations (Table 1). For inhalation exposure, the amount of EOF in descending order was the lungs, lung lavage, liver, kidney, and plasma samples. As for oral gavage, the trend was different from the inhalation exposure with the greatest amount of EOF was detected in the liver, followed by the kidney/plasma, lungs, and lung lavage samples. These trends were also different from target PFOA analysis for both inhalation and oral exposures.

In general, PFOA accounted for over 80% of the EOF in all tissue samples with the exception of plasma samples in the two exposure groups (Table 1). The proportion of PFOA to EOF in plasma samples was lower (around 68% for inhalation and 64% for oral exposure). Mass balance analysis between EOF and target PFOA in these samples indicated the occurrence of potential PFAS metabolites formed by other unknown PFAS precursor and/or other fluorinated compounds that co-existed in the dust samples.

## Discussion

This is, to the best of our knowledge, the first study to measure the bioavailability of PFOA over the lungs following an inhalation exposure to house dust. PFOA was adsorbed to saturation, onto a respirable fraction of house dust collected from residential homes. The use of the PreciseInhale® system proved to be an efficient way of delivering aerosolized house dust to the lung of rats, realistically mimicking dust inhalation exposures and lung deposition. In this study, we compared the bioavailability of PFOA following an inhalation exposure with an oral exposure of dust delivered via gavage. This study reveals that the bioavailability of PFOA over the lung is almost four times higher in blood, at 3 h post exposure, compared to the oral gavage exposure. The present study was experimental without attempts to relate it to neither ambient nor occupational exposure to PFOA.

Hinderliter and co-workers exposed rats via inhalation to PFOA administrated as an aerosolized solution. During the 6-h exposure, the concentration of PFOA in plasma rose proportional (1–25 μg/mL) to the exposure concentration (1–25 mg/m^3^). The C_max_ of PFOA in plasma appeared between 0 and 6 h post the 6-h exposure (Hinderliter et al. 2006). In our study, the C_max_ of PFOA occurred between 0 and 3 h post exposure after which the levels of PFOA in plasma started to decline until 24 h post exposure, a time point where it reached a steady state until the end of the experiment. The time point of termination, 48 h following exposure, was decided based on the results from a pilot study where we observed that the levels of PFOA had started to decline. Our results show that initially within the first 3 h post exposure, the concentration of PFOA was higher from rats that received PFOA via inhalation compared to oral gavage exposure. At 48 h after exposure, the concentrations in plasma following inhalation or oral gavage exposures were similar, although the delivered dose of PFOA was 2.2 times higher via oral exposure. Absorption data following oral exposure to PFOA demonstrated that more than 90% of the delivered dose was absorbed over the gastro intestinal (US EPA 2016; Pizzurro et al. 2019; DeWitt. 2015, kap 6). For inhalation, there is no quantitative estimates of the fractional absorption of PFOA (ATSDR 2021). The absorption process across both the lungs and gut is believed to involve different transporters rather than simple diffusion (US EPA 2016). The transporters organic anion transporters (OATs), organic anion transporting polypeptides (OATPs), and multidrug resistance-associated proteins (MRPs) have been identified to play a role (US EPA 2016). Several of these transporters are known to be expressed in epithelial tissues from the lung and intestine (Roth et al. 2012; Zaïr et al. 2008). The difference in absorption of PFOA between the lung and intestine might be due to different expressions of transporters. Some of the transporters OATs and OATPs are also known to be involved in the excretion of PFOA (Pizzurro et al. 2019). There is a sex difference in urinary excretion of PFOA, where female rats has a greater excretion rate compared to male rats. The transporters OATs and OATPs are expressed in the kidney and are involved in both the excretion into the urine and the reabsorption. Although the mechanism behind this difference in excretion is not fully elucidated, there is some evidence of higher expression of these transporter proteins that are involved in the resorption of chemicals back into the blood in male rats (Pizzurro et al. 2019).

Hinderliter and co-workers also studied sex differences. The elimination of PFOA in females was rapid; hence, at the end of the 6-h exposure time, the levels in plasma were almost twice as high in male compared to the females. The fast elimination in female continued post exposure and was almost complete unlike in males where the elimination of PFOA was slow (Hinderliter et al. 2006). This is in line with the present results, where the plasma concentration in the male rats indicated a slow excretion rate during the 48 h after exposure. There is a large species dependent difference in elimination rate of PFOA where rats have a half-time of a few days compared to humans having a calculated half-time of 2.3–8.5 years (Li et al. 2017). In humans, the long half-time might be associated with a short-term high exposure level of PFOA, while the 8.5 year half-time might be associated with a chronic low exposure to PFOA as well as transformation of other PFOA precursors (Seals et al. 2011). Other study showed that biliary clearance exceeds urinary clearance and both clearance pathways are important (Pizzurro et al. 2019). In another study, only a slight difference in elimination rate of PFOA between the sexes has been observed based on the study group had been exposed to drinking water contaminated with PFAS (Li et al. 2018).

The mechanisms for the bioaccessibility and bioavailability of inhaled SVOCs are complex and have recently been described in Wei et al. (2018, 2020). With bioaccessibility, we here refer to the fraction of a compound that is released into the body fluid and is available for absorption (Caboche et al. 2011; Collins et al. 2015; Rostami and Juhasz 2011; Wei et al. 2018). Further, we acknowledge the definition of bioavailablity as the property of the compound to reach the systemic circulation by crossing the pertinent biological membranes (Collins et al. 2015; Rostami and Juhasz 2011; Yu et al. 2012; Wei et al. 2018). The results from Hinderliter and colleagues indicate that the bioavailability of PFOA over the air-blood barrier is high. Comparing the bioavailability of PFOA exposed either via inhalation as wet aerosol or adsorbed onto particles should imply a simpler mechanism of bioavailability of PFOA delivered as wet aerosol. In our study, the high concentrations of PFOA in the lung tissue and lung lavage indicated that dust-associated and/or desorbed PFOA still remained in the lung compartment at 48 h after exposure.

Regarding organ distribution of PFOA post exposure, there is an almost 2.5 times higher distribution of PFOA to the liver and kidney following inhalation exposures compared to oral gavage. Following an oral exposure, bioavailable substances enter the liver directly via the portal vein. Previous studies have shown that PFOA binds to proteins in blood (Beesoon and Martin 2015; Butenhoff et al. 2012; Forsthuber et al. 2020) and more than 90% of PFOA was typically determined to be bound to albumin in serum (Han et al. 2003). In this study, the ratio of PFOA concentration between liver and serum following an oral exposure to PFOA on dust (364 ± 37 μg/kg bw) was determined to 4.89 at 48 h post exposure. Compared to another study performed by Iwabuchi et al., their ratio was lower (1.67) following an oral exposure to PFOA (100 μg/kg bw) in male rats. The ratio was calculated at Cmax, which was determined to 12 h after exposure. In the same study, the calculated kidney to serum ratio was 0.82 which is also lower compared to that of the current study (1.72) (Iwabuchi et al. 2017). Although 2.2 times lower dose was given to the inhalation exposure, similar liver to serum ratios (5.28) were noted. The tissue distribution of PFOA following different doses has previously shown to be different in rats (male) study where they administrated PFOA intravenously (Kudo et al. 2007). A study on oral exposure to PFOA in rats showed that the tissue distribution was the highest in the liver (Kim et al. 2016). Based on studies with various species, the liver appears to be the dominant tissue for PFAS distribution (Pizzurro et al. 2019). The tissue distribution of radiolabelled PFOA (^14^C-PFOA) in mice following a dietary exposure showed that concentrations were the highest in the liver, followed by the blood, lungs, and kidneys (Bogdanska et al. 2020). The present study demonstrated that following oral exposure, the highest concentrations of PFOA were found in the liver, followed by the kidneys, lungs (lung + lung lavage), and blood.

The disposition of PFOA following exposures differs between rats and humans. In rats, the main distribution of PFOA is to the blood, liver, and kidney of which female rats distributed higher levels of PFOA to the kidney compared to male rats (DeWitt. 2015). In humans, the distribution of PFOA in the body occurred primarily to bone followed by the lungs, liver, and kidney (Pérez et al. 2013). Maestri and colleagues detected the highest levels in the lungs, kidney, liver, and blood, while the neuro system had the lowest levels (Maestri et al. 2006). Since PFOA is not subjected to biotransformation or metabolism in the body, the major elimination route is via excretion (DeWitt. 2015; Stahl et al. 2011).

Overall, most results in the literature show that the major source of exposure to PFOA is via food (Haug et al. 2011a; Poothong et al. 2020; Sunderland et al. 2019; Trudel et al. 2008). However, Haug and colleagues also showed that a large variation in serum concentrations may be related to indoor contamination, because only a smaller variation of the dietary intake in the study group was found. The indoor environment contributed to more than 40% of the PFOA exposure for some people in the study group (Haug et al. 2011a). The indoor environment can be an important factor when characterizing human exposure to PFAS like PFOA (Haug et al. 2011a).

In a previous study, we estimated that the deposited mass of house dust following inhalation is relatively low (Weiss et al. 2018). Yet, the low deposition of dust in the airways is of concern since PFOA is persistent and bioaccumulative.

The absorption kinetics in the lungs for indoor air pollutants has not been subjected to extensive research. In this study, we used house dust, a common occurrence in indoor environments, as a matrix for exposure to air pollution. Chemicals with the property of being SVOCs adsorb to surfaces of particulates, hence house dust may serve as a sink for environmental indoor pollutants (Butte and Heinzow 2002). Previous studies have demonstrated a large spectrum of environmental air pollutants adsorbed to the surface of house dust, including flame-retardants, plasticizers, antioxidants, and PFAS (Eriksson and Kärrman 2015; Mercier et al. 2011; Weschler and Nazaroff 2008). There are scarce experimental data regarding the physicochemical properties of PFAS. The vapor pressure for the PFAS family varies greatly between substances, some of which are within the range of SVOC (Eichler and Little 2020). Evidently, both the predicted and measured vapor pressure of PFOA exceed the upper range of what is defined as SVOCs (Eichler and Little 2020). Nevertheless, several studies provide ample evidence of PFAS presence on house dust from the indoor environment (Eriksson and Kärrman 2015; Weiss et al. 2021; Winkens et al. 2018; Yao et al. 2018). Eriksson and Kärrman (2015) also detected the presence of different classes of PFAS in house dust, and one single group of compounds was measured in levels up to μg/g (Eriksson and Kärrman 2015). In the current investigation, the measured PFOA concentrations explained a majority (65–87%) of the EOF levels in the samples, suggesting that PFOA was the major PFAS coated onto the dust. It should be noted that the imbalance in the ratio of PFOA to EOF in the samples implies the presence of unidentified organofluorine compounds which might be the results of the following scenarios: (1) the dust samples might contain some precursor compounds that can have been metabolized into unknown intermediates; for examples, polyfluoroalkyl phosphate esters (PAPs), a known PFCA precursors that were shown to form PFCAs with a number of unknown degradation intermediates; (2) there were other unidentified PFAS already present in the dust samples, and (3) the PFOA compound might have formed some unknown conjugated products. However, the identification and possible accumulation patterns of such unknown organofluorine compounds are out of the scope of the present work.

In this study, we chose to intubate the rats and deliver the dust directly to the lungs by using the intratracheal inhalation module of the PreciseInhale platform. In this way, we avoided the typical high deposition of such aerosols in the nasal airways that otherwise would have occurred if the more common choice of the nose-only inhalation module had been selected. Bypassing the nasal airways in rodents using intratracheal inhalation is a particular advantage when dealing with aerosols, as in this case, having MMADs toward the upper limit of the respirable interval. From the MPPD aerosol deposition software, it can be calculated that if the same lung-deposited dose in rats would have been required using the nose-only module, 75% would have been caught in the nasal airways, leaving only 25% in the target area of the lower respiratory tract. Another advantage of using intratracheal exposures is the avoidance of a dominating fraction of inhaled dust initially depositing in the nose, but subsequently redistributing to the gastro-intestinal tract by mucociliary clearence. Because of the high aerosol concentration during the inhalation exposures, it is likely that the dominating fraction of PFOA remained on the particulate fraction instead of desorbing to the gas phase. Another advantage with the high dose rate is that even a dose target of 0.5 mg particles inhaled can be reached in approximately 20 min, which is a particular advantage during kinetic studies. The active dosing mechanism of the PreciseInhale helps to keep the standard deviation between repeated exposures generally below ±10–15% (Fig. 2). The house dust spiked with PFOA represents another complex powder substrate that can be prepared and aerosolized for small scale inhalation exposures using the Dustgun generator of the PreciseInhale® platform. Previously, powders such as diesel soot, silica dust, and palladium nanoparticles have been generated into respirable aerosols (Ewing et al. 2006; Gerde et al. 2004; Wilkinson et al. 2011).

In conclusion, we demonstrated that there is a fast absorption over the airways to the systemic circulation of dust-associated PFOA, following inhalation exposures to house dust. The concentration of PFOA in plasma is approximately four times higher compared to the same deposition level of exposure via oral gavage. The ubiquitous presence of PFOA in indoor environment and exposures of humans is of concern, due to the long half-times of duration in humans. Several endocrine related effects have been shown following exposure to PFOA, and important target tissues are mammary gland, thyroid, and adipose tissue (DeWitt. 2015). It is known that PFOA pass the placenta into the fetal circulation (DeWitt. 2015). At the stage of development from infant to the prepubertal period, there are key moments in development that are sensitive to chemicals, which may cause long term effects (Birnbaum and Fenton 2003). The observations presented in this manuscript emphasize house dust as a potentially important matrix of exposure to indoor pollution. The investigation of indoor pollutant bioavailability, via either inhalation, oral, or skin absorption is largely unexplored and requires further research.

## Data Availability

All data generated or analyzed during this study are with the corresponding author, and, if necessary, she is available for taking any question about the datasets and these can be requested by reasonable request.
